# Skeletal adaptation to mechanical cues during homeostasis and repair: the niche, cells, and molecular signaling

**DOI:** 10.3389/fphys.2023.1233920

**Published:** 2023-10-17

**Authors:** Pablo J. Atria, Alesha B. Castillo

**Affiliations:** ^1^ Department of Orthopedic Surgery, New York University Grossman School of Medicine, New York, NY, United States; ^2^ Department of Biomedical Engineering, New York University Tandon School of Engineering, New York, NY, United States

**Keywords:** skeletal stem and progenitor cells, SSPCs, bone, mechanical loading, mechanical signals, fracture repair, niche

## Abstract

Bones constantly change and adapt to physical stress throughout a person’s life. Mechanical signals are important regulators of bone remodeling and repair by activating skeletal stem and progenitor cells (SSPCs) to proliferate and differentiate into bone-forming osteoblasts using molecular signaling mechanisms not yet fully understood. SSPCs reside in a dynamic specialized microenvironment called the *niche*, where external signals integrate to influence cell maintenance, behavior and fate determination. The nature of the niche in bone, including its cellular and extracellular makeup and regulatory molecular signals, is not completely understood. The mechanisms by which the niche, with all of its components and complexity, is modulated by mechanical signals during homeostasis and repair are virtually unknown. This review summarizes the current view of the cells and signals involved in mechanical adaptation of bone during homeostasis and repair, with an emphasis on identifying novel targets for the prevention and treatment of age-related bone loss and hard-to-heal fractures.

## 1 Introduction

The skeleton plays a crucial mechanical role in our daily lives by facilitating movement, providing support against gravitational forces, acting as an endocrine organ and protecting internal organs against blunt force trauma (Castillo and Leucht, 2015). The ability of bones to adapt and respond to the prevailing mechanical environment over one’s lifetime is critical for maintaining skeletal health, mineral homeostasis and meeting mechanical demands of everyday activities (e.g., walking, running, jumping, etc.) (Li and Xie, 2005; Chen et al., 2013; Castillo and Leucht, 2015; Cabahug-Zuckerman et al., 2020).

Regular physical activity and exercise can stimulate bone growth and increase bone density, thereby reducing the risk of fracture. However, with aging and disease (e.g., rickets, Paget’s disease, diabetes, malignancy, etc.) (Augat et al., 2005; Heilmeier et al., 2016), there is a diminishment in bones’ ability to adapt to mechanical stress over time (Morgan et al., 2018), leading to bone fragility and increased fracture risk. One critical contribution to bony non-union is delayed or inhibited revascularization of the injury site, revascularization depends on appropriate biological and mechanical cues, and recent data suggest that osteoprogenitor (OPC)-endothelial cell (EC) crosstalk, playing a critical role in revascularization of the injury site (Kusumbe et al., 2014; Yang et al., 2022; Biswas et al., 2023). Skeletal stem and progenitor cells (SSPCs) play a vital role in maintaining bone mass and repairing damaged bones. SSPCs reside in a specialized microenvironment known as the niche which acts as the central hub for maintaining cellular identity during quiescence and coordinating a response to mechanical and biological signals. In bone, SSPCs have been found in the periosteum, endosteum, marrow and growth plate (Méndez-Ferrer et al., 2010; Zhou et al., 2014; Coutu et al., 2017; Debnath et al., 2018; Matsushita et al., 2020a; Kurenkova et al., 2020).

Current FDA approved anabolic treatments that can prevent bone loss are Teriparatide, Abaloparatide and Romosozumab. The first two are PTH analogs, while Romosozumab is a sclerostin inhibitor. All of these medications suppress bone remodeling, and might have an effect on the cellular populations which line the bone surface (Leaffer et al., 1995; Hodsman et al., 2005), even though this process has not been fully understood. Therefore, understanding the mechanisms involved in SSPC niche regulation is crucial for developing therapeutic strategies to prevent and treat skeletal disease and injury.

This review focuses on the identity of murine SSPCs, their unique environment in different bone compartments, and their involvement in bone homeostasis and repair. We then describe the mechanical environment in bone, relying heavily on previous comprehensive reviews by the senior author, with emphasis placed on the interplay between the niche, SSPCs and their response to mechanical signals during homeostasis and repair.

## 2 Bone compartments and their skeletal stem and progenitor cells

Stem cells are defined as cells with the ability to (Castillo and Leucht, 2015) reconstitute an environment that supports hematopoiesis (Li and Xie, 2005); self-renew on the clonal level; and (Cabahug-Zuckerman et al., 2020) differentiate into multiple lineages (Wagner et al., 2005). SSPCs include skeletal stem cells and downstream progenitors and are located in the niche the periosteum, endosteum and within bone marrow (Bianco et al., 2001; Méndez-Ferrer et al., 2010; Zhou et al., 2014; Shi et al., 2017; Debnath et al., 2018; Duchamp de Lageneste et al., 2018; Seike et al., 2018; Ortinau et al., 2019; Matsushita et al., 2020b; Shen et al., 2021). However, the extent to which distinct SSPC populations contribute to bone repair is still a matter of debate, largely due to the lack of proper markers to distinguish between the different populations. To date, SSPC populations have been characterized using a variety of markers such as Mx1, Grem1, LepR, Cxcl12, Pdgfra, Pdgfrb and Prrx1, among others (Table 1). Additionally, only a handful of studies have made quantitative comparisons of the contribution of uniquely identified SSPC populations to bone repair, making it difficult to compare results between studies (Matsushita et al., 2020b; Shen et al., 2021; Jeffery et al., 2022). State-of-the-art technologies, such as single cell RNA-seq and spatial transcriptomics have helped elucidate transcriptional characteristics of different bone resident cell populations, but none of the aforementioned markers is restricted to a single population, making it challenging to investigate their distinct functions during skeletal growth, repair, aging and adaptation (Baccin et al., 2020).

**TABLE 1:** Markers and mouse lines labeling SSPCs in injury.

Marker		Location	Type of injury	Potential contribution to bone repair	Pathway
LepR Zhou et al. (2014)	*LepR-cre*	Periosteum	Monocortical injury		
Cxcl12 Matsushita et al. (2020b)	*Cxcl12-creER*	Bone marrow (perisinusoidal)	Monocortical injury	Differentiate into mature osteoblasts	Wnt/B-catenin signaling
Adipoq Zhong et al. (2020)	*Adipoq:Td*	Bone marrow	None	Unknown	Unknown
Adipoq Jeffery et al. (2022)	*Adipoq-cre*	Bone marrow	Monocortical injury	Proliferation, differentiation into mature osteoblasts	Unknown
Oln Shen et al. (2021)	*Oln* ^*iCreER*^	Bone marrow (periarteriolar)	None (just mechanical stimulation)	Unknown	Unknown
Prrx1 Duchamp de Lageneste et al. (2018)	*Prx1-Cre;mTmG*	Bone marrow and periosteum	Bicortical		Periostin
Gli1 Jeffery et al. (2022)	*GlicreER* ^*T2*^	Periosteum	Bicortical	Proliferation, differentiation into mature osteoblasts	Wnt/β-catenin
Gli1 Shi et al. (2017)	*Gli1-CreER* ^*T2*^ *; Ai9*	Bone marrow and Periosteum	Bicortical	Proliferation and differentiation	
Fgfr3 Matsushita et al. (2023)	*Fgfr3-creER*	Endosteum	Monocortical injury	Expand and differentiate to osteoblasts in young bones	Wnt/B-catenin signaling
Mx1, aSMA Ortinau et al. (2019)	*Mx1-Cre;aSMA-GFP*	Periosteum	Monocortical injury	Supply the majority of callus-forming cells	
Pdgfra Julien et al. (2022)	*Pdgfra* ^*CreERT*^	Various tissues	Bicortical		BMP signalign
Ctsk Debnath et al. (2018)	*CTSK–mGFP*	Periosteum (marks also osteoclasts)	Bicortical	Proliferation, osteoblast differentiation	

The most primitive SSPCs have reticular morphology and can be identified by leptin receptor (LepR) expression (Zhou et al., 2014). They also express high levels of CXC motif chemokine ligand 12 (Cxcl12) (Matsushita et al., 2020b) and stem cell factor (Scf), key factors maintaining the hematopoietic stem cell niche, hematopoietic stem cells (HSCs) and restricted progenitors (Zhou et al., 2014). This subpopulations will be discussed in details throughout this review.

SSPCs can originate from different bone compartments and even from adjacent skeletal muscle. Prx1+ SSPCs, a population that resides in the periosteum, bone marrow, and skeletal muscle, can form cartilage, adipose tissue and bone during bone healing (Julien et al., 2021). Lineage tracing and scRNA-seq showed that Prx1+ periosteal cells and mesenchymal progenitors in skeletal muscle are enriched in osteochondral progenitors, and contribute to endochondral ossification during fracture repair. Both populations transition to a fibrogenic state prior to chondrogenesis which is activated by BMP signaling (Sivaraj et al., 2021).

**FIGURE 1:**
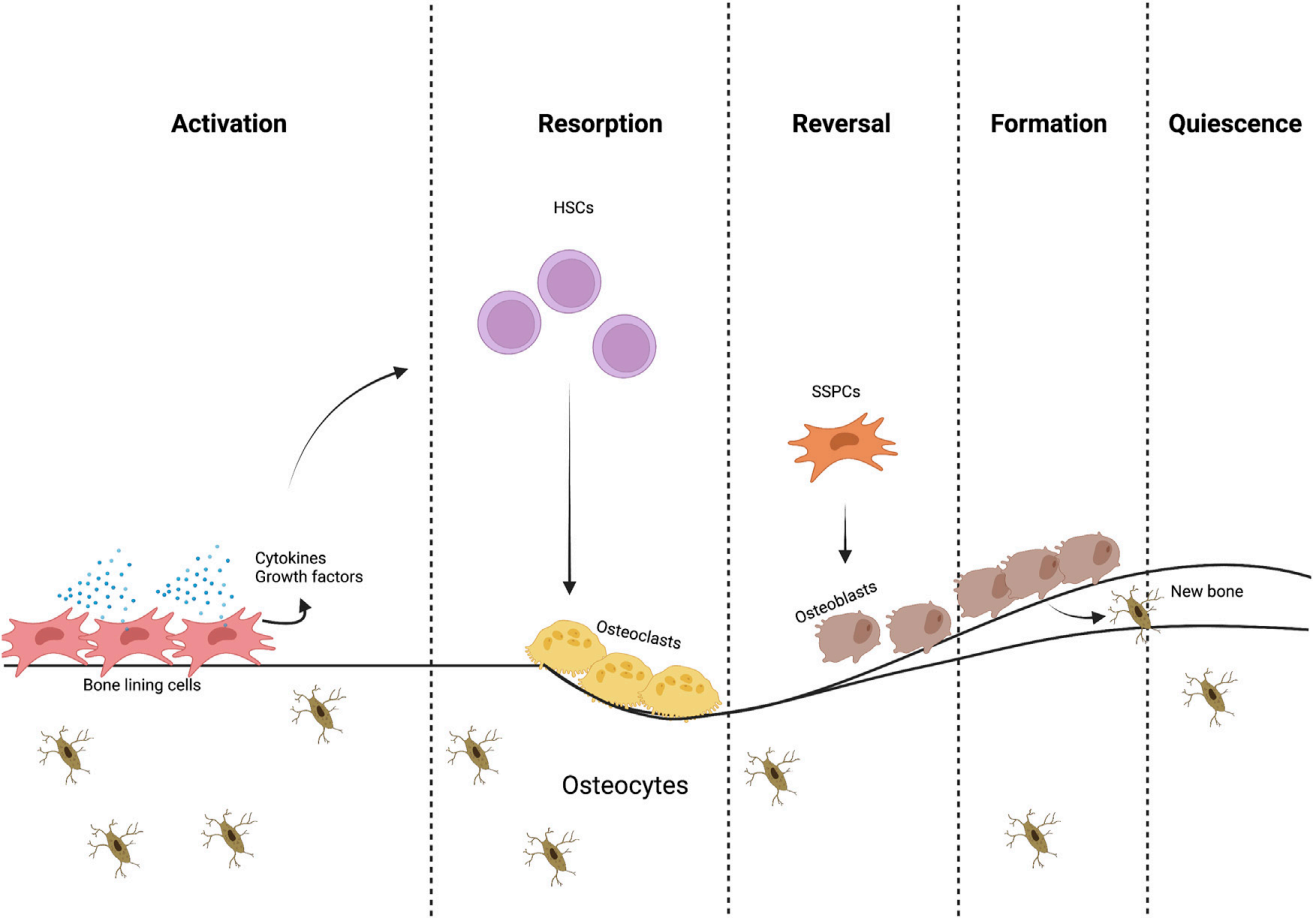
The bone remodeling process. Different cells and signaling molecules involved in the bone remodeling process, bone resorption by osteoclasts and formation by osteoblasts.

Cellular niches are dynamic microenvironments consisting of cellular and extracellular elements that regulate maintenance, self-renewal and differentiation of stem cells (Li and Xie, 2005; Kurenkova et al., 2020). These niches exist in different bone compartments (periosteal, endosteal and marrow), with the marrow containing trabecular bone in both metaphyseal and epiphyseal compartments. These different niches are influenced by a variety of metabolic products; for example, calcium and reactive oxygen species, have been shown to have a direct influence in stem cell behavior (Ito et al., 2004). Regarding mechanical stimulation, the response to mechanical cues in these distinct environments differs due to their unique makeup of cells and stroma (connective tissue, blood vessels, lymphatic vessels, and nerves) and calcified tissues of varying microstructure, which determines their mechanical properties (Robling et al., 2006; Gurkan and Akkus, 2008; Petzold and Gentleman, 2021). Presumably, each compartment contains distinct niches that vary in SSPC identity and heterogeneity. In the last year, there has been significant progress towards understanding the diversity of stromal cell populations owing to single-cell RNA seq and spatial transcriptomics (Baccin et al., 2020) (Figure 1).

**FIGURE 2:**
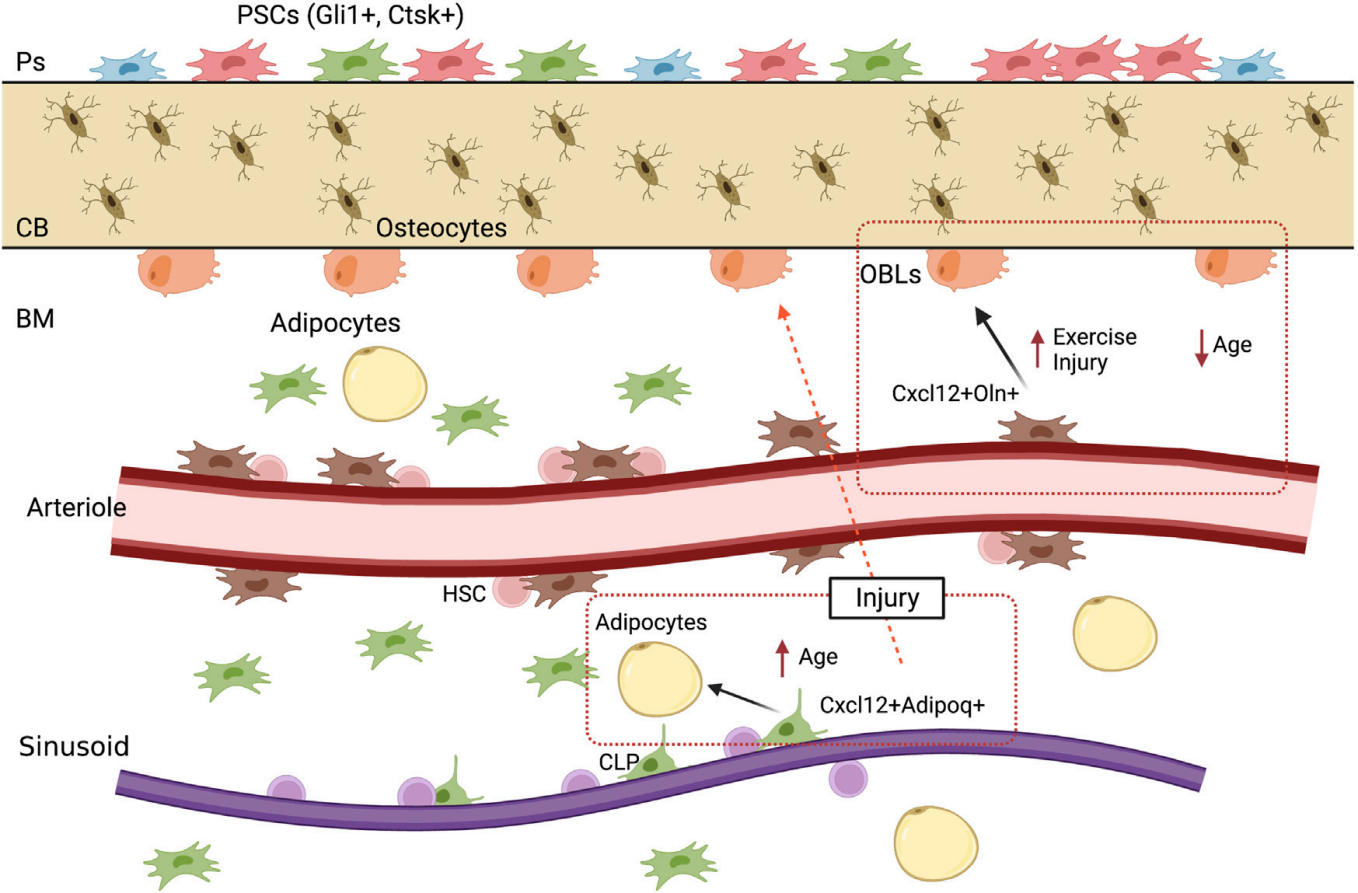
Bone compartments and their SSPCs. Something similar to Figure 1 generated before, the difference is that now it will describe periosteum, endosteum and bone marrow.

Characterizing the location and composition of these niches, as well as understanding their response to mechanical signals and injury is important for developing effective therapeutic strategies to prevent and treat osteoporosis and fractures that are difficult to repair (Estell and Rosen, 2021). What is known presently is described below (Figure 2).

### 2.1 Periosteum

The periosteum is a thin external membrane of connective tissue that covers bones, it is composed of two layers: the outer fibrous layer and the inner cambium layer. The cambium layer contains stem and progenitor cells with chondrogenic and osteogenic capacity, which has been described elsewhere. (Lazzeri et al., 2009). Several markers, including Sca1, α-SMA, Prx1, Mx1, Ctsk, have been used to identify stem and progenitor population in the periosteum (Debnath et al., 2018; Duchamp de Lageneste et al., 2018; Ortinau et al., 2019; Matthews et al., 2021). Periosteal stem cells can regenerate bone tissues even in absence of bone marrow, which highlights their importance (Ortinau et al., 2019). Recently, a Ctsk+CD200+ population has been identified as periosteal stem cells (Debnath et al., 2018). This population can differentiate into osteogenic lineage cells, as well as into chondrocytes; however, Ctsk + cells do not express LepR (Colnot, 2009; Debnath et al., 2018). Rather, LepR + cells in the periosteum overlap with Gli1+ periosteal cells (Jeffery et al., 2022). Indeed, recent data show that in the adult periosteum, Gli1^creERT2^ expression identifies periosteal SSPCs, while marrow SSPCs are identified by LepR^cre^ and Adiponectin-cre/creER expression. Following bone injuries, both Gli1-creER+ and LepR + cells exhibit proliferation but contribute differently to the bone repair process (Jeffery et al., 2022). Gli1+ cells in the periosteum mainly contribute to endochondral ossification after bicortical fractures and give rise to bone marrow stromal cells residing in a perivascular niche after losing the expression of *Gli1* and acquiring expression of *LepR*, *Scf*, and *Cxcl12* (Jeffery et al., 2022). How these unique populations respond to mechanical cues both during homeostasis and fracture repair remains unknown.

### 2.2 Endosteum

During appositional bone growth, the endosteum is formed by the periosteum becoming trapped. The endosteum is a thin membrane, typically measuring only 10–40 µm in thickness, consisting of a loosely defined layer of connective tissue and a small number of cell layers.

The cells within the endosteum are arranged in a mosaic pattern, with formative, resting, and resorptive regions characterized by the presence of active osteoblasts, preosteoblasts, or osteoclasts, respectively (Frost, 1987). In terms of function, the endosteum contributes significantly to bone repair and reconstruction, as it houses osteoprogenitor cells like MSCs and preosteoblasts, much like the periosteum. The endosteum has been widely studied due to its importance as the site for hematopoietic stem cells (HSC) niche, and the characteristics of HSC compared to their central marrow counterparts (Haylock et al., 2007). It has been shown that HSCs residing in the endosteal region have different proliferative capacity and homing efficiency compared to central HSCs, highlighting the influence of site-specific niches (Sicl et al., 2013). SSPC niches are believed to exist in the metaphysis and endosteum, given the presence of cells expressing SSPC markers such as GLI family zinc finger 1 (Gli1), Gremlin 1 (Grem1), Leptin receptor (LepR), Nestin-GFP, Platelet-derived growth factor receptor a (PDGFRa), and PDGFRb. Recently, Matsushita et al. (2023) identified a novel SSPC population, which highly expresses *Fgfr3,* this population possesses osteoblast-chondrocyte transitional identity and diminishes with age.

However, the characteristics of these SSPC populations in the endosteum are not well-defined (Loopmans et al., 2022).

### 2.3 Marrow

The bone marrow contains hematopoietic stem cells (HSCs) which engage in hematopoiesis throughout the entire adult life. LepR+ and Cxcl12+ SSCs that are contained within the bone marrow space are essential components of the HSCs niche, due to the fact that they secrete essential factors for HSC maintenance (Zhou et al., 2014). Osteoblasts, are also important for the maintenance of the niche and some restricted progenitors, as they also provide important factors (Lévesque et al., 2010). In young and middle-aged C57BL/6 J mice, the percentage of LepR + cells in total bone marrow cells was reported to be between 0.7% and 11% (Kara et al., 2023). In postnatal mice, LepR + cells recovered 95% and 85% of all CFU-Fs from the bone marrow and femur shaft, respectively (Shu et al., 2021). Numerous single-cell RNA sequencing based studies have shown that LepR, Cxcl12 and Adipoq are expressed by the same cells in the adult bone marrow (Baryawno et al., 2019; Tikhonova et al., 2019; Matsushita et al., 2020b). Adipoq + cells are perivascular and are distributed throughout the bone marrow with similar location to LepR + cells (Jeffery et al., 2022). It has been shown that these Adipoq + cells do not contain lipid droplets, form a 3D network within the marrow space, and are essential in maintaining bone marrow vasculature, as well as playing an important role in regulating bone formation (Zhong et al., 2020).

If we analyze what has been reported regarding bone marrow SSPCs, LepR + largely overlap with Cxcl12+ cells (Zhou et al., 2014), this LepR + Cxcl12+ population could be divided into two different populations according to their specific location; LepR + Cxcl12+ periarteriolar cells and LepR + Cxcl12+ perisinusoidal cells (Baccin et al., 2020). It has been shown that LepR + cells that locate surrounding arterioles, can be further identified by the expression of Oln (Shen et al., 2021), this population is mechanosensitive, which means that is maintained by mechanical stimulation, as well as it has the ability to differentiate into mature osteoblasts (Shen et al., 2021). Additionally, perisinusoidal Cxcl12+ cells, are a quiescent cell population which are primed to become adipocytes, although, under special conditions can differentiate into mature osteoblast, this population also expresses Adipoq (Matsushita et al., 2020b).

Sivaraj and colleagues reported that bone marrow stromal cells (MSCs), which fall under the SSPC umbrella, found in the metaphysis (mpMSCs) and diaphysis (dpMSCs) are unique, that is, mpMSCs are PDGFRα^+^ *β* ^+^Hey1^+^ while dpMSCs are PDGFRα^+^ *β* ^+^Hey1^−^, mpMSC can be efficiently differentiated to osteogenic, adipogenic, and chondrogenic lineage cells *in vitro,* and can also give rise to dpMSCs during bone development (Sivaraj et al., 2021). This highlights the substantial heterogeneity among MSCs, and illustrates the fundamental differences between distinct locations and microenvironments.

Besides both perivascular populations, it has also been identified a non-perivascular population with *in vivo* osteogenic and chondrogenic potential labeled by *Grem1,* although their contribution to adult bone is limited (Worthley et al., 2015).

## 3 Mechanical environment in bone

The skeleton is composed of cortical and trabecular bony architectures, differing both in mechanical characteristics and metabolic activity. The manner in which these tissues amalgamate to form complete bones is crucial in determining the overall mechanical properties of the organ. Additionally, factors such as size, shape, and cross-sectional area of the bone significantly influence its properties, and these features can be altered due to age-related changes or disease processes (Morgan et al., 2018). Differences between cortical and trabecular bone are mainly dictated by tissue porosity. Cortical bone has a porosity of 5%–15%, while trabecular bone has a porosity of 40%–95%. Cortical bone exhibits anisotropic behavior; that is, the longitudinal direction of the cortical bone, which is aligned with the diaphyseal axis, has greater strength and tensile/compressive modulus compared to the radial and circumferential directions (Morgan et al., 2018). Mechanical properties of trabecular bone at the apparent level - the level at which several trabeculae are observed at once - are mainly influenced by its porosity. Trabecular bone exhibits higher strength in compression compared to tension and is weakest in shear, although these variations diminish with decreasing apparent density. A more comprehensive review of this topic is found in Morgan, E. F., et al. (2018). “Bone mechanical properties in healthy and diseased states.” (Morgan et al., 2018).

Bone adapts to mechanical cues as part of its homeostatic program. Physical activity, which transmits mechanical forces to the tissue, sends mechanical signals that affect cells at a molecular level, changing their gene expression, proliferation, differentiation, and apoptosis (Jacobs et al., 2010). Without these signals, bone undergoes increased resorption which translates into tissue loss. These changes in bone mass and architecture due to mechanical loading and unloading are described by a theory termed “the mechanostat” (Frost, 1987). The mechanostat theory classifies bone behavior based on mechanical strain and models the effect of influences on the skeleton through effector cells, osteocytes, osteoblasts, and osteoclasts (Frost, 1987).

Osteocytes are the most abundant cells in bone tissue, dispersed throughout the mineralized matrix, with their lacuna-canalicular system and dendritic connections, are the primary mechanosensors, mechanotransducers and major producers of some signaling proteins (Palumbo and Ferretti, 2021), able to detect metabolic changes, as well as detect and transmit mechanical cues to downstream signals that regulate bone cell activity. They can sense mechanical forces such as hydrostatic pressure, fluid shear stress, and direct deformation and convert them into biochemical and biological signaling events. This conversion involves four different elements: force transmission to cells, mechanosensing, signal transduction, and signal transmission (Carina et al., 2020). Specifically, SSPCs, osteoblasts, chondrocytes, and endothelial cells can respond directly to mechanical signals. Two recent reviews summarize molecular mechanisms underlying the transduction of mechanical cues into biochemical signals (Chen et al., 2013; Castillo and Leucht, 2015; Anani and Castillo, 2022).

The bone anabolic threshold refers to the minimum level of mechanical strain or deformation required to stimulate new bone formation. This threshold varies depending on a number of factors including age, sex, and genetic variability. If the strain magnitude exceeds the minimum strain threshold, bone formation is activated in those regions experiencing increased. The anabolic strain threshold (>1,050 microstrain) for initiating new bone formation *in vivo* (Turner et al., 1994) and for activating mechanoresponsive signaling pathways in bone cells (>10,000 microstrain) (You et al., 2000) has been estimated. During walking, tissue-level deformation or strain on bone surfaces can vary between 500 and 2,000 microstrain (Martelli et al., 2014), while strenuous activity can result in strains up to 10,000 microstrain (Milgrom et al., 2000). Whole bone strain plays a crucial role in facilitating fluid flow within the bony matrix, lacuna-canalicular space, and marrow (Piekarski and Munro, 1977; Birmingham et al., 2015). Additionally, fluid drag at cell attachment points along the osteocyte processes can amplify these strains, leading to osteocyte membrane strains estimated to be up to 30,000 microstrain (Verbruggen et al., 2012).

The fundamental principles governing the response of healthy, uninjured bone to mechanical signals have been established through seminal studies conducted both *in vivo* and *in vitro*, as reviewed in (34). These include (Castillo and Leucht, 2015): bone responds to dynamic loading (Li and Xie, 2005); bone responds only after distinct strain or strain rate thresholds are crossed (Cabahug-Zuckerman et al., 2020); the bone formation response correlates with strain magnitude and rate (Chen et al., 2013); bone responds to short loading periods (Augat et al., 2005); bone grows accustomed to routine mechanical signals (Heilmeier et al., 2016); bone is highly responsive to mechanical signals during growth and development (Morgan et al., 2018); aging results in a dysregulated bone response to mechanical signals (Castillo and Leucht, 2015). While these principles are important to consider and to think about, they do not explain the events that are occurring at the niche level, which means, understanding the SSPCs involved in the response, which autocrine or paracrine signals are involved in this response, and how different locations affect this response.

## 4 SSPCs in mechanoadaptation of bone

Riffault et al. (2020) investigated the effect of mechanical loading on bone marrow stromal/stem cells using LepR-cre; tdTomato + animals. *In vivo* axial compressive loading of the tibia did not result in proliferation of LepR-cre; tdTomato + stromal cells within the marrow or in the recruitment of these cells to the bone surface. The finding that LepR + cells did not significantly contribute to bone formation in adult mice is not unexpected, as previous research has shown that these cells only make up a small proportion of Col2.3+ cells in 2-month-old mice (3%–10%) and 10-month-old mice (10%–23%), with LepR + osteocytes only appearing at 10 months of age (Zhou et al., 2014). Instead, it suggests that these cells may play a supportive role in osteogenesis via cell non-autonomous effects or that LepR + cells already present along the bone surface are reactivated.

As mentioned before, Shen et al., showed that a specific LepR + subpopulation, which expresses exclusively Oln+, are located in the bone marrow, specifically in the peri-arteriolar niche, which is mechanosensitive. The peri-arteriolar niche contains unique cell populations that promote the growth and differentiation of both bone-forming cells and immune cells, specifically the LepR + Oln + cells, which are shown to be maintained by physical exercise, and their depletion directly affects the common lymphoid progenitor population, by decreasing its number. With regard to mechanism, removing Piezo1, a mechanosensitive ion channel protein (Ma et al., 2022), from Oln + cells led to lower bone mineral density, as well as reduced frequencies of Oln + cells and CLPs. Additionally, Piezo1 deletion resulted in a weakened response to sudden infection, which could be attributed to the close connection between Oln + cells and CLP (Shen et al., 2021).

Prrx1+ cells are primarily located in the periosteum and play a significant role in bone repair (Liu et al., 2019). Periosteal progenitors are a source for osteoblasts and become osteocytes in response to mechanical loading via a primary cilium-mediated process, but the exact mechanism is yet to be confirmed (Moore et al., 2019). The acute response of adult bone to loading involves expansion of Sca‐1+Prrx1+ and Sca‐1−Prrx1+ cells in the periosteum (Cabahug-Zuckerman et al., 2019). Both adult and aged mice exhibit load-induced periosteal bone formation, though the response is significantly attenuated with age (Cabahug-Zuckerman et al., 2019). The Sca-1+Prrx1+ population is targeted by loading, and loading activates proliferation of Prrx1+ cells in the periosteum as early as 2 days into a 4-consecutive-day loading protocol. Prrx1+ cells may play a key role in load-induced osteogenesis considering their presence in the periosteum, the primary site of load-induced cortical bone formation (Cabahug-Zuckerman et al., 2019). However, further research is needed to fully understand the role of Prrx1+ cells in load-induced bone formation.

Interestingly, recent studies seem to suggest that the origin of mature osteoblasts and adipocytes in homeostasis shifts between young (P21) and adult mice (18 M), they specifically identified a shift from Fgfr3+ cells to LepR + cells with age, which raises the question if the SSPCs population(s) involved in load-induce bone formation also undergoes this point of origin change (Matsushita et al., 2023).

In a separate study, Osx + cells or their progeny accounted for >98% of periosteal cells at sites of bone formation (Zannit and Silva, 2019). Approximately 30% of Osx + lineage cells arose via proliferation, and a recent study by the same group showed that ablation of proliferating osteoblast reduces lamellar bone formation, demonstrating that proliferating cells are necessary to achieve a maximal anabolic response to mechanical loading (Zannit et al., 2020). While these data suggest that recruitment and differentiation of more primitive osteoprogenitors is not required for the early response to acute anabolic loading, the origin and turnover of these periosteal-resident Osx + cells are still unclear.

## 5 SSPCs in bone repair

Jeffery and others (Jeffery et al., 2022) observed that periosteal SSPCs could be identified by Gli1creERT2 expression, whereas SSPCs in marrow were identified by LepR-cre and Adiponectin-cre/creER expression. After bone injuries, both SSPC populations underwent proliferation but contributed differently to the bone repair process. Gli1+ periosteal SSPCs were found to mainly contribute to endochondral ossification after bicortical fractures and gave rise to marrow SSPCs that lost Gli1 expression and acquired a perivascular localization with expression of LepR, Scf, and Cxcl12. In contrast, LepR + Adipoq + cells only contributed to intramembranous repair. These findings underscore the distinctions between the two populations and their respective microenvironments (Jeffery et al., 2022).

LepR + Adipoq + cells, which are mainly found surrounding sinusoids and are fated to become adipocytes unless under specific conditions such as bone injury. These Adipoq + cells have distinct molecular signatures and respond differently to different types of signals compared to other SSPC populations; this cell population, which has been also referred as *MALPs*, has been shown to be critical for bone marrow regulation, including vasculature and bone formation (Zhong et al., 2020). It has been shown that ablating this Adipoq + population decreases the number of Emcn^+^CD31^+^ endothelial cells, as well as causing an increase in trabecular bone formation. Adipoq + cells have an important regulatory role since are the cell population that expresses *Csf1* the most, which encodes the macrophage colony-stimulating factor (M-CSF); this factor is paramount in the proliferation, differentiation, survival and function of myeloid lineage cells, including monocytes, macrophages, and osteoclasts (Inoue et al., 2023; Zhong et al., 2023).

As mentioned before, Jeffery et al. have shown that LepR + Adipoq + cells are located exclusively in the bone marrow compartment, are responsible for adult steady-state osteogenesis and actively participate in drill-hole injuries, which mean, injuries that heal via intramembranous repair (Jeffery et al., 2022).

Matsushita et al. (2020b) found that a specific type of quiescent bone marrow stem cell, marked by Cxcl12-creER, which correspond to perisinusoidal LepR + cells, can transition into a precursor cell state similar to skeletal stem cells during injury responses mediated by canonical Wnt signaling. These cells contribute to skeletal regeneration but do not participate in cortical bone osteoblast formation under homeostasis. Taken into consideration previous research, and the data from Matsushita et al. we believe that this Cxcl12-creER population corresponds to the LepR + Adipoq+ and MALPs population.

## 6 Summary and future approaches

**FIGURE 3:**
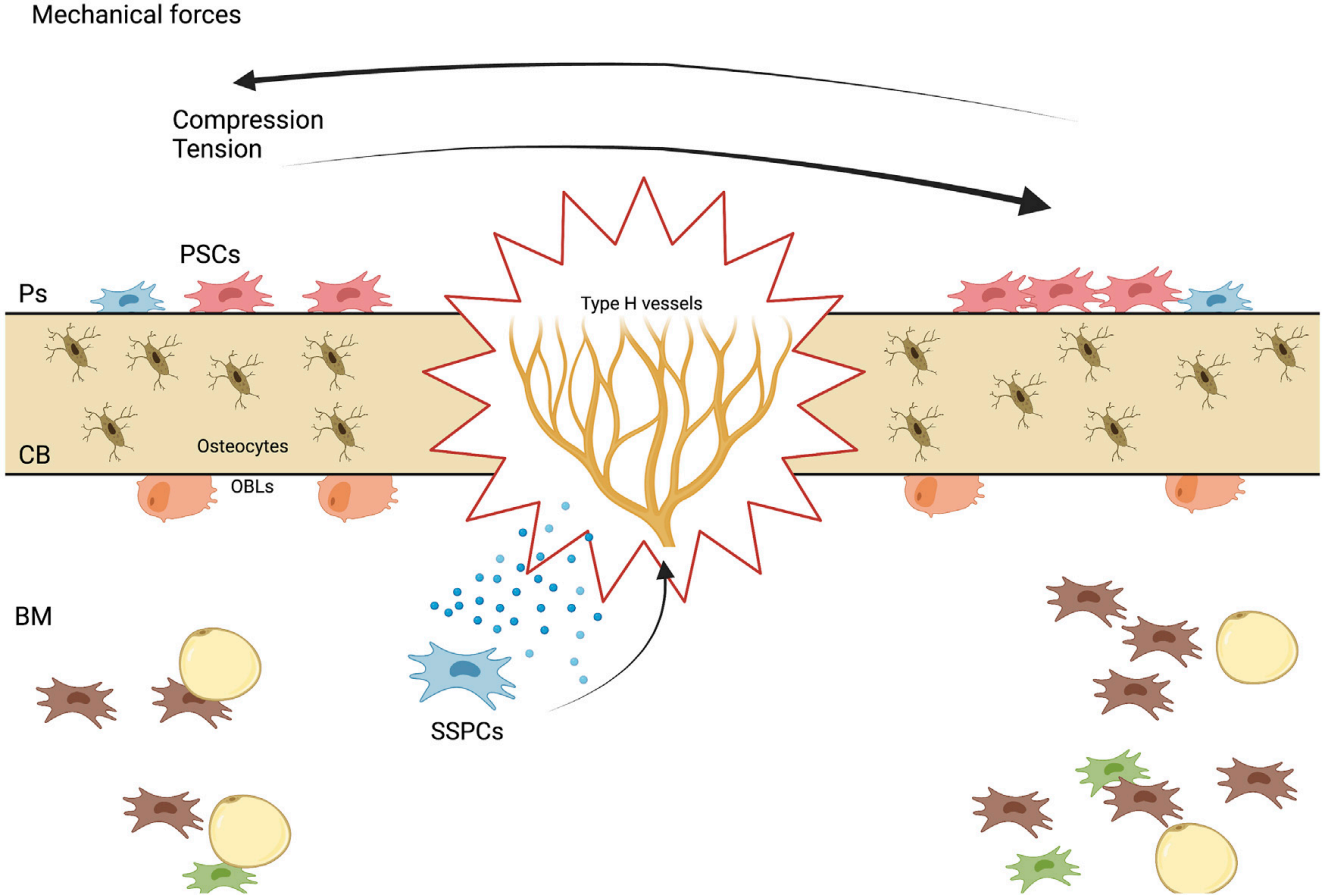
Mechanisms of stem cell-mediated bone regeneration. A diagram showing the various mechanisms by which stem cells promote bone regeneration, including differentiation into bone-forming cells, paracrine signaling to stimulate endogenous repair processes, and immunomodulatory effects.

As it was described, load induced bone formation during homeostasis and repair is a complex process which encompasses many biological events, which involve a variety of growth factors, the activation of niche specific SSPCs, differentiation and activation of osteolineage cells such as osteoblasts and osteoclasts, angiogenesis, among others (Figure 3).

The first need is to try to understand which SSPCs population or populations are involved in load-induced bone formation. This involvement can be either by activation and differentiation into mature osteoblasts, or it might be that some of these populations are acting as regulatory paracrine networks, providing the necessary signals and growth factors to either quiescent bone lining cells, stromal cells, or others. Whether these osteoblasts derive from one or several different sources remains to be elucidated.

We consider that the identification of more upstream therapeutic targets is relevant in injury and bone loss, due to the fact that it has been described, for aged individuals, that the SSPCs pool population declines with age; therefore, identifying potential factors that could aim to maintain the number and functionality of this multipotent cell populations might grant clinicians different treatment options depending on the clinical scenario.
